# Use of the Cascade expandable net to treat cerebral vasospasm – initial clinical experience from a single centre with in vitro benchside tests

**DOI:** 10.1186/s42155-021-00275-x

**Published:** 2021-12-08

**Authors:** P. Bhogal, T. Simpanen, K. Wong, D. Bushi, M. A. Sirakov, S. Sirakov, M. Aggour, L. Makalanda

**Affiliations:** 1 Department of Interventional Neuroradiology, The Royal London Hospital, Barts NHS Trust, Whitechapel Road, London, E1 1BB UK; 2 Department of Radiology, The Royal London Hospital, Barts NHS Trust, Whitechapel Road, London, E1 1BB UK; 3 Perflow Medical, 4 Hatzoran St., 4250604 Netanya, Israel; 4 Department of Interventional Neuroradiology, University Hospital St. Ivan Rilski, Sofia, Bulgaria

**Keywords:** Vasospasm, Aneurysm, Cascade, Stentplasty

## Abstract

**Background:**

The use of self-expanding stents to treat post-hemorrhagic cerebral vasospasm was recently described. We sought to determine the clinical efficacy of the Cascade device to treat delayed cerebral vasospasm (DCV). We performed benchside tests to determine the chronic outward force exerted by the Cascade in comparison to the Solitaire.

**Methods:**

The chronic outward force (COF) of the Cascade M agile and Cascade L Agile was tested with equivalent tests of the Solitaire 4x20mm. Further tests to determine the forces generated in pre-formed tubes of 1.5–6 mm were performed using both fully and partially unsheathed Cascades.

A retrospective review to identify all patients with aSAH and DCV treated with a Cascade device between January 2020 and July 2021. We recorded the treatment arterial vessel diameters and hemorrhagic or ischemic complications.

**Results:**

In vitro the Cascade generated greater radial force than the Solitaire. The force generated by the Cascade M Agile at 1.5 mm was approximately 64% higher than the Solitaire 6x40mm and approximately 350% higher than the Solitaire 4x20mm.

4 patients with DCV were identified all of whom were treated with a cascade device. In all cases there was a significant improvement in the diameter of the vasospastic vessels treated with an average diameter increase of approximately 300%. There were no complications from the Cascade. Delayed CT angiography showed persistent dilatation of the segments treated with the Cascade at 24 h.

**Conclusion:**

The Cascade is a safe and effective device when used to treat DCV secondary to aSAH. Larger studies are required to validate our initial results.

## Introduction

Delayed cerebral vasospasm is an important cause of delayed neurological deterioration in patients with aneurysmal subarachnoid haemorrhage (aSAH) (Kassell et al., 1990; Kassell et al., 1985). Angiographic vasospasm is an independent predictor of poor outcome following aSAH and there is a strong correlation between the severity of vasospasm and the incidence of cerebral infarction, which itself has a strong association with poor outcome following SAH (Crowley et al., 2011; Vergouwen et al., 2011a; Vergouwen et al., 2011b; Fergusen & Macdonald, 2007). The severity of vasospasm correlates with the degree of hypoperfusion and hence the majority of the delayed infarcts are related to severe vasospasm (Brown et al., 2013; Vatter et al., 2011; Weidauer et al., 2007; Dankbaar et al., 2009; Dhar & Diringer, 2015).

Triple-H therapy has been recommended however euvolaemia is now considered optimal. Although hypertension is widely used a recent randomised controlled trial (RCT) showed an increase in side effects and no long-term improvement clinically (Gathier et al., 2018). Endovascular treatments represent an alternative strategy and typically involve intra-arterial (IA) vasodilators and/or balloon angioplasty. Recently, stent-retrievers and stent like devices have been used to successfully treat cerebral vasospasm (Bhogal et al., 2017a; Bhogal et al., 2017b; Kwon et al., 2019; Badger et al., 2020; Su et al., 2020).

We present the first published cases using the Cascade device to treat cerebral vasospasm following aSAH. We describe the angiographic results in four patients as well as the in vitro bench side tests employed to determine the radial forces developed by the Cascade.

## Methods

### Cascade device

The Cascade is a non-occlusive fully retrievable neck-bridging support device designed to provide temporary support during coil embolization of intracranial aneurysms. The device received CE mark in late 2018.

The Cascade is constructed from 42 braided nitinol and platinum wires that form a compliant net-like structure. The platinum wires enable good visualisation of the device. A short, flexible distal wire, and radio-opaque markers are positioned at the proximal and distal ends of the braided parts of the device. The original devices can be delivered through a 0.021-in. microcatheter and are available in two diameter sizes with the Cascade M recommended for vessel diameters of 2-4 mm and the Cascade L recommended for vessels 4-6 mm in diameter. The Agile models are shorter versions of the M and L devices. More recently, a smaller model - Cascade-17, compatible with 0.017-in. microcatheters was added, thus a total of five models are available.

The device is delivered in compressed form via the microcatheter and subsequently inflated and deflated via the handle. The control handle has two modes – auto-lock mode for stepwise radial expansion and continuous mode for smoother and more gradual expansion that can also provide tactile feedback. In auto-lock mode each ‘click’ of the handle results in controlled expansion of the device. The device can be fully deployed or partially deployed if it is only partially unsheathed.

### In vitro analysis

#### Chronic outward force testing

**Fig. 1:**
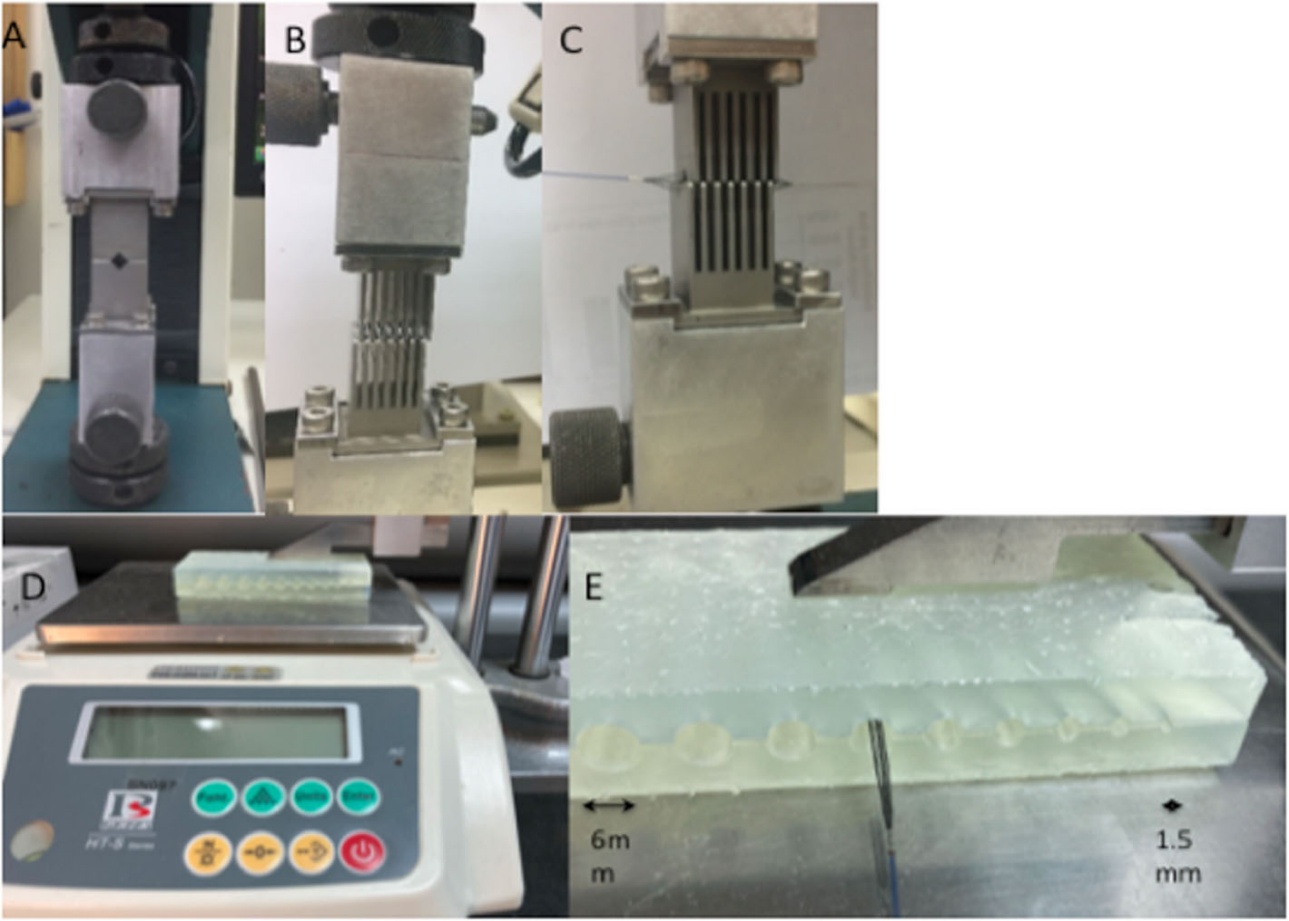
The H5KT (Tinius Olsen, Horsham, PA, USA) tensile machine connected to two interlocking combed plates (Fig. 1**A** and **B**) was used to determine the COF. Each of the devices were placed into the aperture created by the two interlocking plates Fig. 1**C**). Device set-up to test the force generated by the different devices when inserted into ‘cylinders’ of varying diameter is shown in Figs. 1**D** and **E**

The chronic outward force (COF) of the Cascade M agile and Cascade L Agile was tested with equivalent tests of the Solitaire 4x20mm. The COF testing was performed using a H5KT (Tinius Olsen, Horsham, PA, USA) tensile machine connected to two interlocking combed plates (Fig. 1A and B). Each device, in their fully expanded state, was placed into the aperture created by the two interlocking plates. Bringing the two plates together slowly decreased the diameter of the aperture. The COF was measured for each device at various different diameters between 1.5 mm (maximal compression) and 6 mm (unconstrained) (Fig. 1C).

In addition to calculating the COF we sought to determine the radial force applied by the device per ‘click’. For Cascade L Agile and Cascade M Agile, the expansile forces of the Cascade mesh per handle click were measured using the experimental set-up shown in Fig. 1D and E. Two complementary box shapes blocks were printed using a 3D printer. One surface of each of the block includes half cylinder cross-section channels with varied diameters ranging from 1.5 to 6 mm. One of the blocks was attached to the surface of an electronic balance and the second block was attached to height gauge (Fig. 1D). Using the height gauge, the upper block was slowly lowered toward the block on the electronic balance until just made contact but did not apply any pressure to the scale. This set-up allowed the construction of a series of ‘tubes’ with pre-determined internal diameters (1.5 to 6 mm) that could simulate different vessel diameters thought to represent normal and vasospastic vessels (Fig. 1E). Each Cascade device was inserted in its collapsed position, into each tube (Fig. 1E). The mesh was opened in auto-lock mode and the expansile forces of the Cascade per handle click were measured. The test was repeated with the Solitaire 4 × 20 mm and Solitaire 6 × 40 mm by positioning each Solitaire device into each tube. In this second series of experiments the Cascade devices were also partially unsheathed (50% unsheathed) to determine the forces generated when the device was deployed in this state.

### Clinical

#### Patient selection

We performed a retrospective review to identify all patients with aSAH who developed cerebral vasospasm and were treated with the Cascade device between January 2020 and July 2021.

### Endovascular treatment

Treatments were performed under general anaesthesia via the right common femoral/radial artery using a 6Fr system with standard heparin anticoagulation (5000 IU) to maintain the activated clotting time between 2 and 2.5 times above baseline. After angiography an 0,021 or 0,027 in. microcatheter was tracked into the vasculature distal to the vasospasm segment followed by careful unsheathing of the Cascade device under continuous fluoroscopic guidance. The choice of using the Cascade L or M to treat the vasospasm was at the discretion of the operator however, in general the larger device – Cascade L was chosen if the ICA required stentplasty treatment and either the Cascade M /Cascade M Agile or Cascade 17 was chosen for segments distal to the ICA termination. The device was expanded several times and total expansion was under 5 min in all cases. In cases of residual focal stenosis post stentplasty the Cascade device was partially unsheathed in order to focus the dilatationary effect. Intra-arterial vasodilators, were not injected prior to the stentplasty treatment however if there was persistent vasospasm of the distal vasculature (M3 or A3 segments and beyond) then chemical vasodilators were injected slowly.

### Angiographic measurement of treatment response

The percent-fold changes in vessel diameter were calculated as a proportional change in vessel diameter post-angioplasty and pre-angioplasty compared with the vessel diameter pre-angioplasty. Vessel diameter was measured at the most stenotic portion of vasospasm.

## Results

### Clinical cases

#### Case 1

**Fig. 2:**
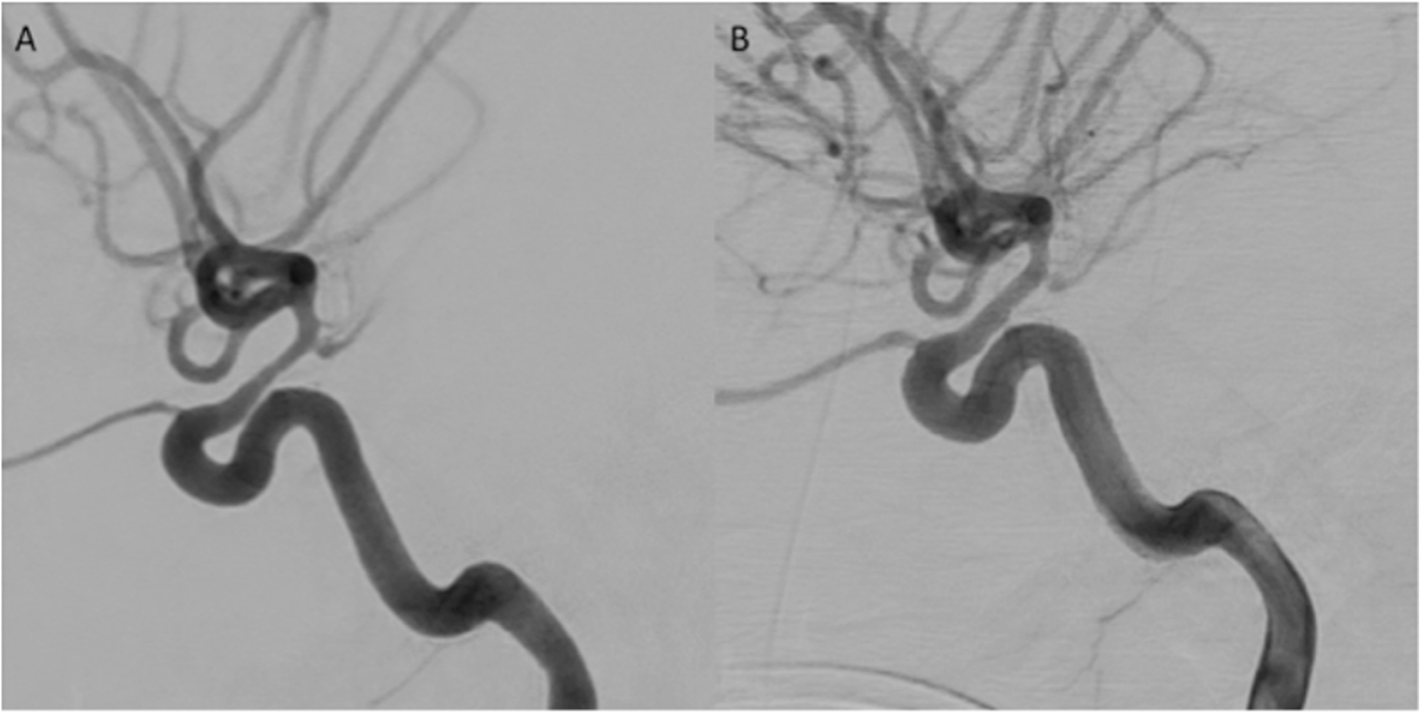
Lateral angiograms of the terminal ICA demonstrating stenosis pre-treatment (**A**) and post-dilation (**B**)

A 24-year old male patient presented with a ruptured partially thrombosed, giant, right terminal ICA and MCA aneurysm (WFNS grade 5, Fisher 3). During clipping the aneurysm re-ruptured necessitating occlusion of the ICA for haemorrhage control that resulted in right MCA infarction and consequent hemi-craniectomy. Two weeks post-ictus he developed new right-sided weakness and CTA demonstrated terminal ICA stenosis. Catheter angiography confirmed moderate/severe stenosis of the terminal ICA (≈65%). Using a Phenom 27 microcatheter (Medtronic), a Cascade L Agile was used for stentplasty. After initially using the device in its fully unsheathed state targeted stenplasty was performed by partially unsheathing the device. The initial pre-treatment vessel diameter was 1.4 mm (4.1 mm pre-vasospasm diameter) (Fig. 2A) and post-dilatation the vessel measured 3.3 mm (Fig. 2B) resulting in a 235% increase in the diameter of the vessel and approximately 20% residual stenosis at the end of the treatment. At 90 days the patients was mRS 6.

#### Case 2

**Fig. 3:**
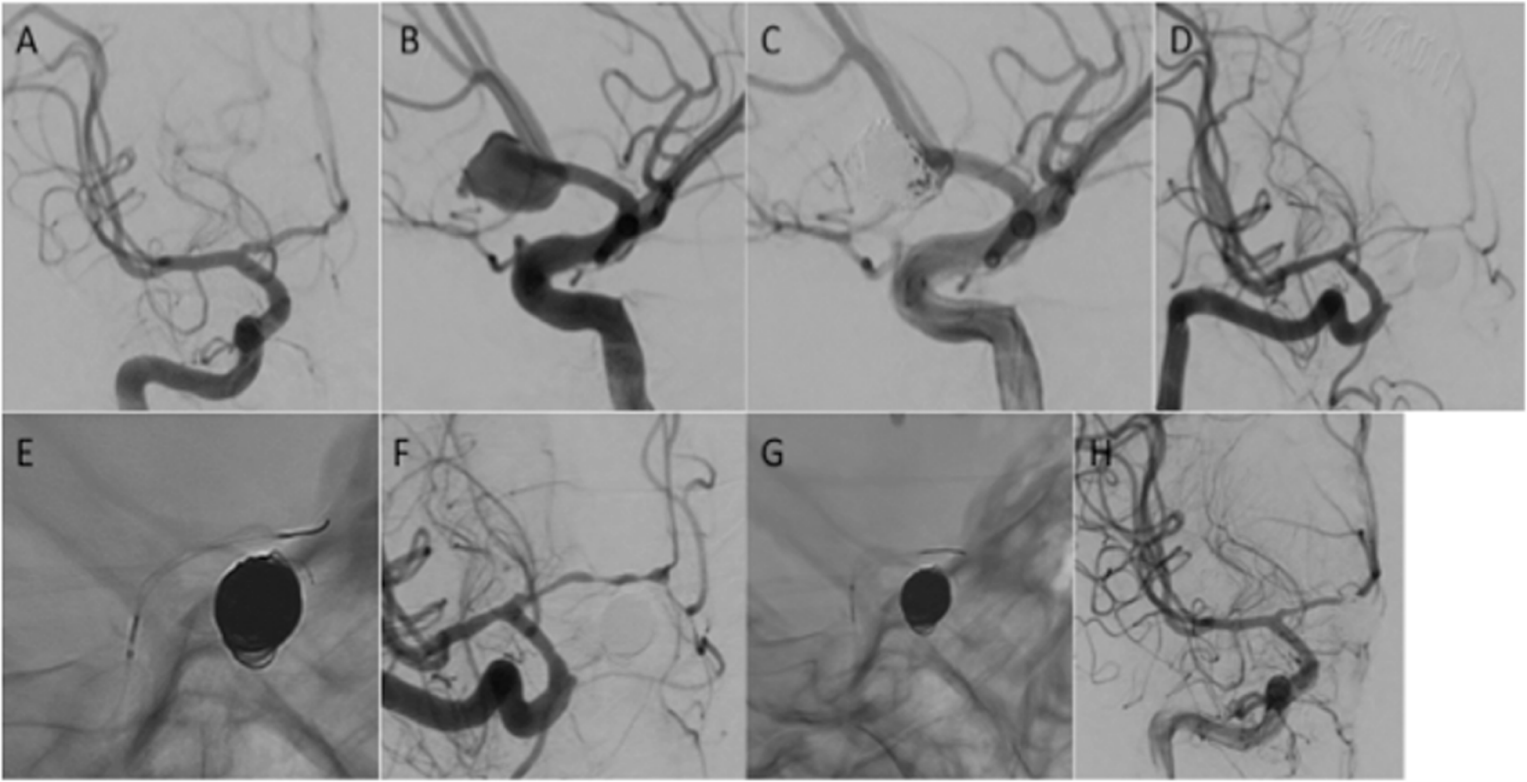
On angiography of the right ICA there was no significant filling of the aneurysm (Fig. 3**A**) that filled mainly via the left A1 segment (Fig. 3**B**). Coil embolisation was performed (Fig. 3**C**). Angiography confirmed severe stenosis of the right A1 (Fig. 3**D**) that was treated with a Cascade M Agile (Fig. 3**E**). After the initial stentplasty there was a significant improvement in the calibre of the A1 segment however, focal stenosis remained (Fig. 3**F**) and treated with the device partially unsheathed (Fig. 3**G**). Angiography at the end of the procedure revealed no significant stenosis and virtually normal appearance of the A1 segment (Fig. 3**H**)

A 43-year old male was admitted with aSAH secondary to rupture of a 15 mm lobulated anterior communicating artery aneurysm (Fig. 3A and B). Coiling was performed with minimal filling of the neck of the aneurysm seen on final angiography (Fig. 3C) with no intra-operative complications. On day 6 the patients GCS decreased. Following emergency CTA, catheter angiography demonstrated severe right ACA stenosis (70%), particularly the A1 segment with delayed filling distally (Fig. 3D). Using a Phenom 21 microcatheter a Cascade M Agile was tracked into the ACA. After the initial expansion of the fully unsheathed device (Fig. 3E) there was good vasodilatation however, focal areas of spasm remained (Fig. 3F) and therefore, the procedure was repeated but with the device partially unsheathed to target the areas of persistent spasm (Fig. 3G). This resulted in complete restoration of the normal calibre of the A1 segment and normalisation of anterograde flow in the distal ACA (Fig. 3H). The pre-treatment vessel diameter was 0.7 mm and post-dilatation the vessel measured 2.2 mm resulting in a 314% increase in the diameter of the A1 segment and normal restoration of the vessel calibre. The patient’s symptoms improved and there was no clinical evidence of recurrent vasospasm.

#### Case 3

**Fig. 4:**
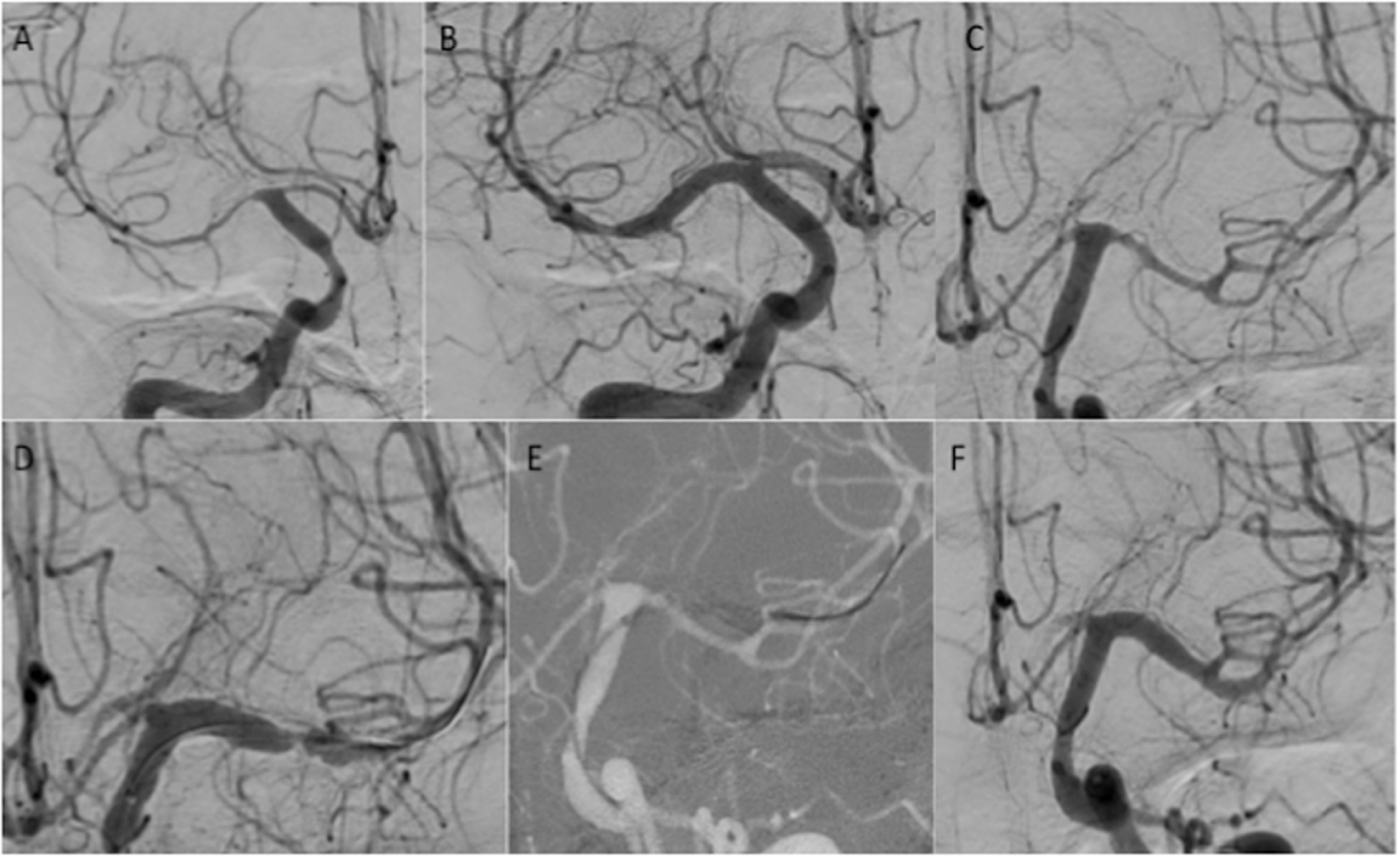
Severe vasospasm of the MCA and ACA was noted on the right (Fig. 4**A**). The right M1 segment was treated with a Cascade L that resulted in normalisation of the calibre (Fig. 4**B**). The severe spasm seen on the left (Fig. 4**C**) was also treated with the Cascade L however, on angiography after the initial expansion of the device persistent focal spasm of the distal M1 segment was seen (Fig. 4**D**) that was treated by partially unsheathing the cascade device to focus the radial force a at this location (Fig. 4**E**) with complete calibre normalisation seen on the final angiography (Fig. 4**F**)

**Fig. 5:**
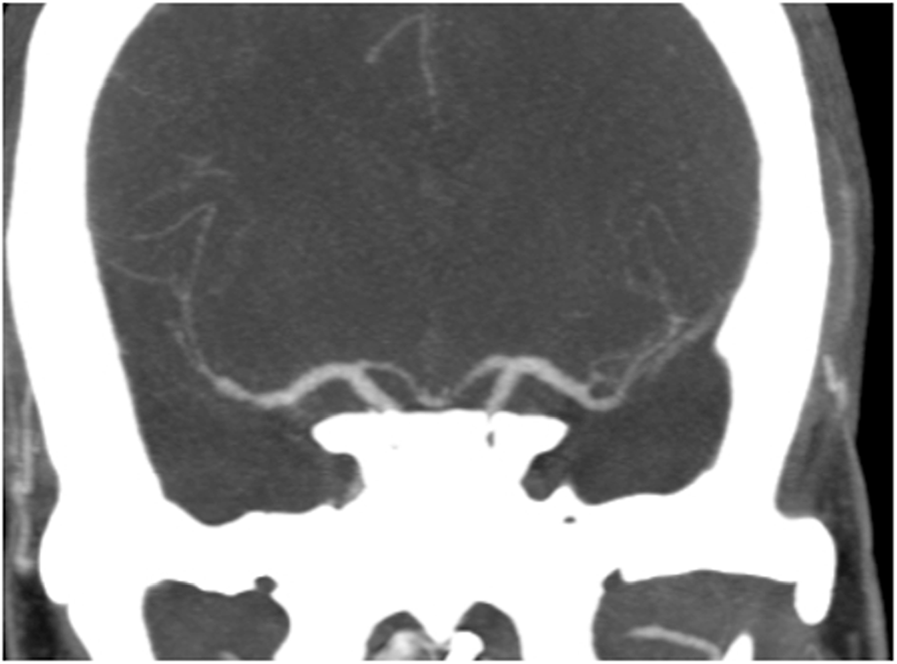
A 24 h CTA showed persistent vasodilatation of the vessel segments treated using the Cascade

A 29-year old female patient was admitted with a ruptured dissecting distal left PICA aneurysm (Fisher 3, WFNS 5) treated with parent vessel occlusion. On day 6 the patient became increasingly hypertensive, and emergency CTA confirmed severe bilateral MCA and ACA vasospasm with further vasospasm affecting the posterior circulation confirmed on catheter angiography (Fig. 4A and C). A Cascade L was used to treat the vasospasm of the right M1 and proximal M2 segments with complete restoration of normal calibre (Fig. 4B). On the left, after the initial expansion of the fully unsheathed device, there was a focal area of stenosis affecting the distal M1 segment (Fig. 4D) treated with a partially unsheathed Cascade (Fig. 4E). This resulted in complete restoration of normal calibre in the M1 and proximal M2 segments with markedly improved flow seen distally (Fig. 4F). On the right the pre-treatment M1 diameter was 0.6 mm and post-dilatation it measured 3.1 mm resulting in a 514% increase in diameter. On the left the pre-treatment M1 diameter was 1.2 mm and post-dilatation the left M1 segment measured 3.7 mm resulting in a 308% increase in diameter. A 24 h CTA demonstrated persistent vasodilation of the treated segments (Fig. 5).

#### Case 4

**Fig. 6:**
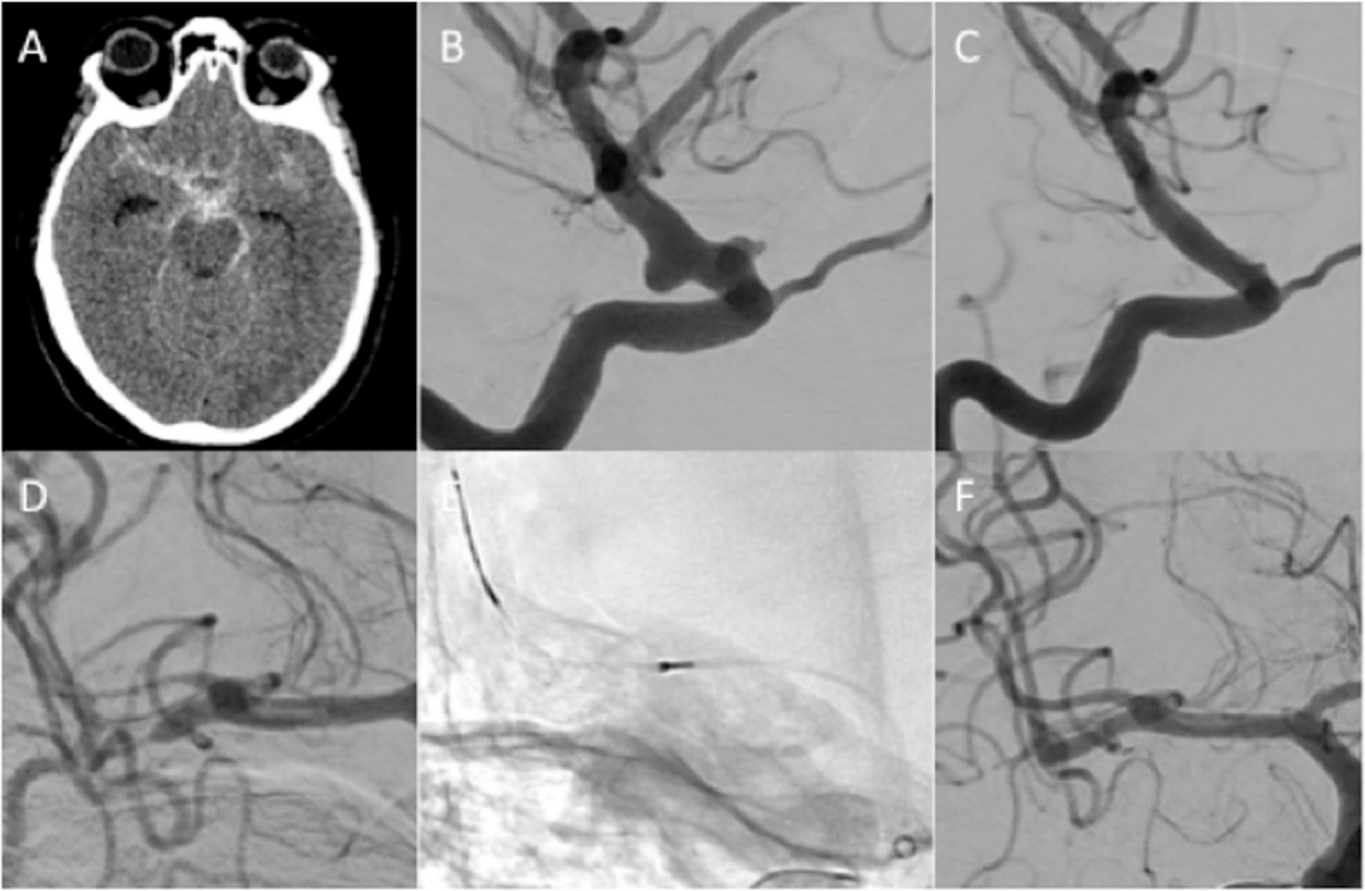
A female patient was admitted with aSAH (Fig. 6**A**). Angiography demonstrated two supraclinoid ICA aneurysms (Fig. 6**B**) treated with flow diversion and coils (Fig. 6**C**). Delayed vasospasm affecting multiple territories developed with severe vasospasm seen affecting the right proximal M2 branches (Fig. 6**D**). Stentplasty using a Cascade 17 (Fig. 6**E**) with no residual stenosis post-procedure (Fig. 6**F**)

A 54 year old female patient was admitted with aSAH (WFNS 2, Fisher 3) (Fig. 6A) and two aneurysms located on the left supraclinoid ICA (Fig. 6B) that were treated using a PED Shield (Medtronic) and coils (Fig. 6C). On day 7 the patient developed worsening left upper-limb weakness with severe bilateral MCA vasospasm seen on CTA and confirmed on catheter angiography. The severe vasospasm of the proximal right M2 branches (Fig. 6D) was successfully treated using a Cascade 17 device (Fig. 6E, F). Thee vasospasm of the left M1 was treated using a Cascade M Agile. The mean diameter of the vasospastic M2 branches was 0.5 mm with the average post-treatment diameter measuring 1.8 mm resulting in a 360% increase in diameter and no residual vasospasm. The vasospastic left M1 segment measured 1 mm and post vasodilation measured 2.2 mm resulting in a 220% increase in the diameter and a 10% residual stenosis. A 24 h-CTA showed persistent vasodilation of the stent-plastied segments.

### Clinical complications

There were no complications related to the Cascade or stentplasty procedure.

### In vitro results

**Fig. 7:**
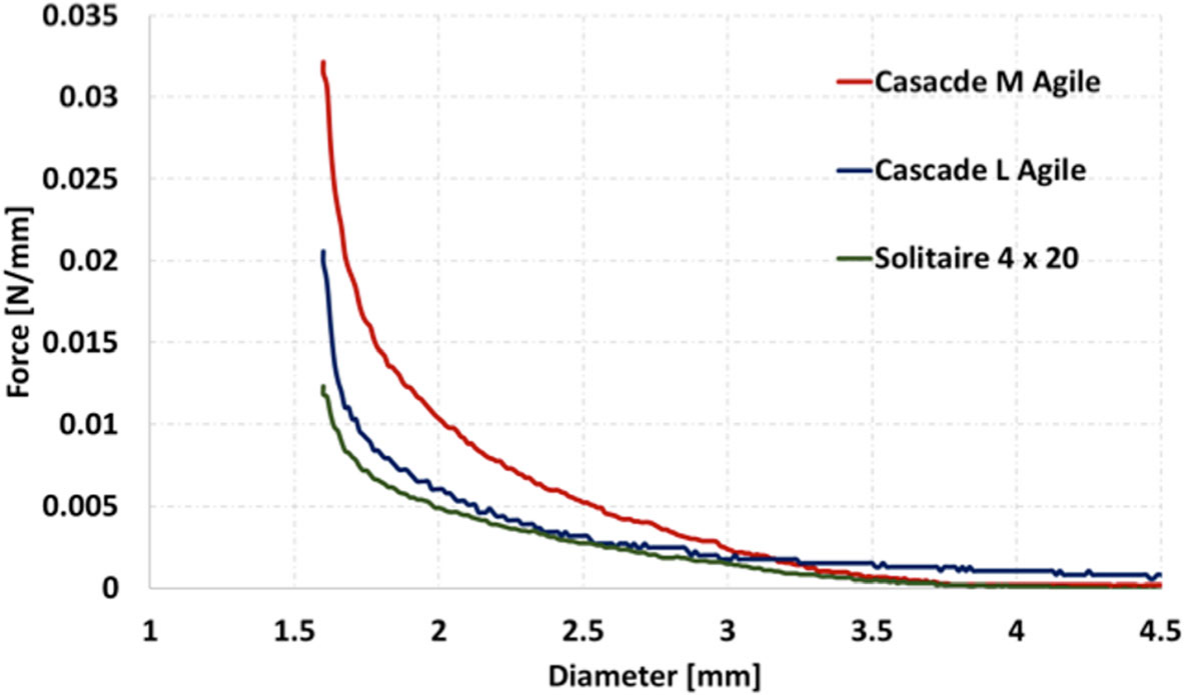
The COF of the Cascade M, L Agile and Solitaire 4x20mm devices. The Cascade M Agile showed persistently higher COF compared to the other devices

The results for the COF with the devices fully unsheathed are shown in Fig. 7. There is a gradual reduction in the COF as all the devices tested are expanded to maximal dimension. Both the M and L Cascade Agile devices generated greater COF compared to the Solitaire 4x20mm device across range of the diameters tested. The Cascade M Agile generated the highest COF of the devices tested and the force generated was greatest at smaller diameters.

**Fig. 8:**
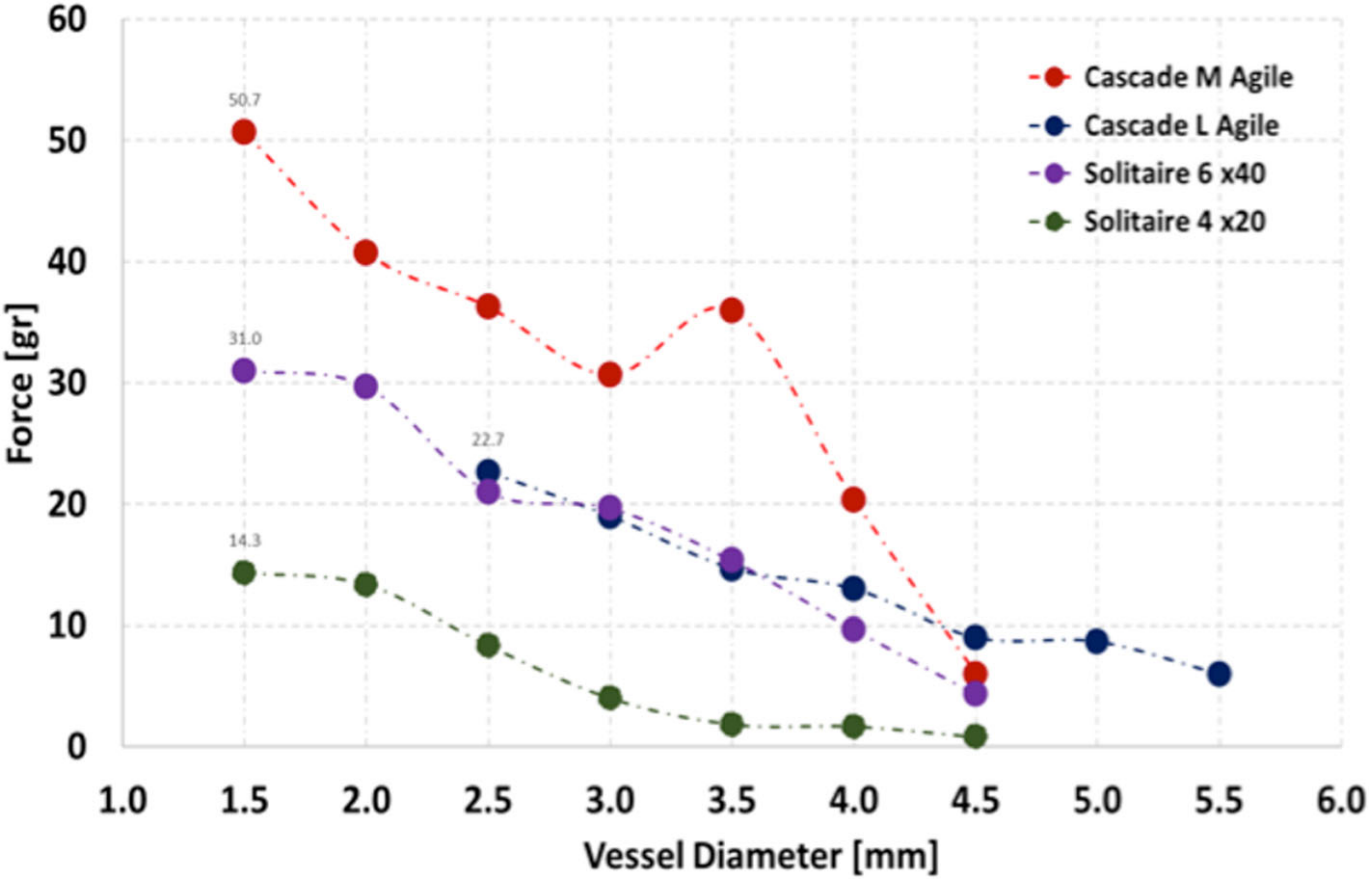
The maximal expansile force generated by each device at different diameters. The Cascade M Agile consistently generated significantly higher forces than either of the Solitaires and the Cascade L Agile. The forces generated by the Cascade devices gradually decreases towards 0 as their unconstrained/maximum diameter is reached

The expansile radial force generated by the devices when placed in the preformed tubes of different diameters is shown in Fig. 8. The Cascade M Agile generated the greatest force when compared to both the Cascade L Agile and both the Solitaire 4x20mm and 6x40mm devices. The force generated by the Cascade M Agile at 1.5 mm was approximately 64% higher than the Solitaire 6x40mm and approximately 350% higher than the Solitaire 4x20mm. The force generated remained consistently higher for the Cascade M Agile device compared to both Solitaire devices across all diameters that may be relevant for vasospastic vessels. The force generated by the Cascade L Agile was similar to the Solitaire 6 mm device at small diameters however, at larger diameters the force generated by the Cascade L Agile remained consistently higher. It is important to note that these were the maximum forces able to be generated by the cascade devices and lower force could also be generated by limiting the degree of device expansion.

### Partial unsheathing

**Fig. 9:**
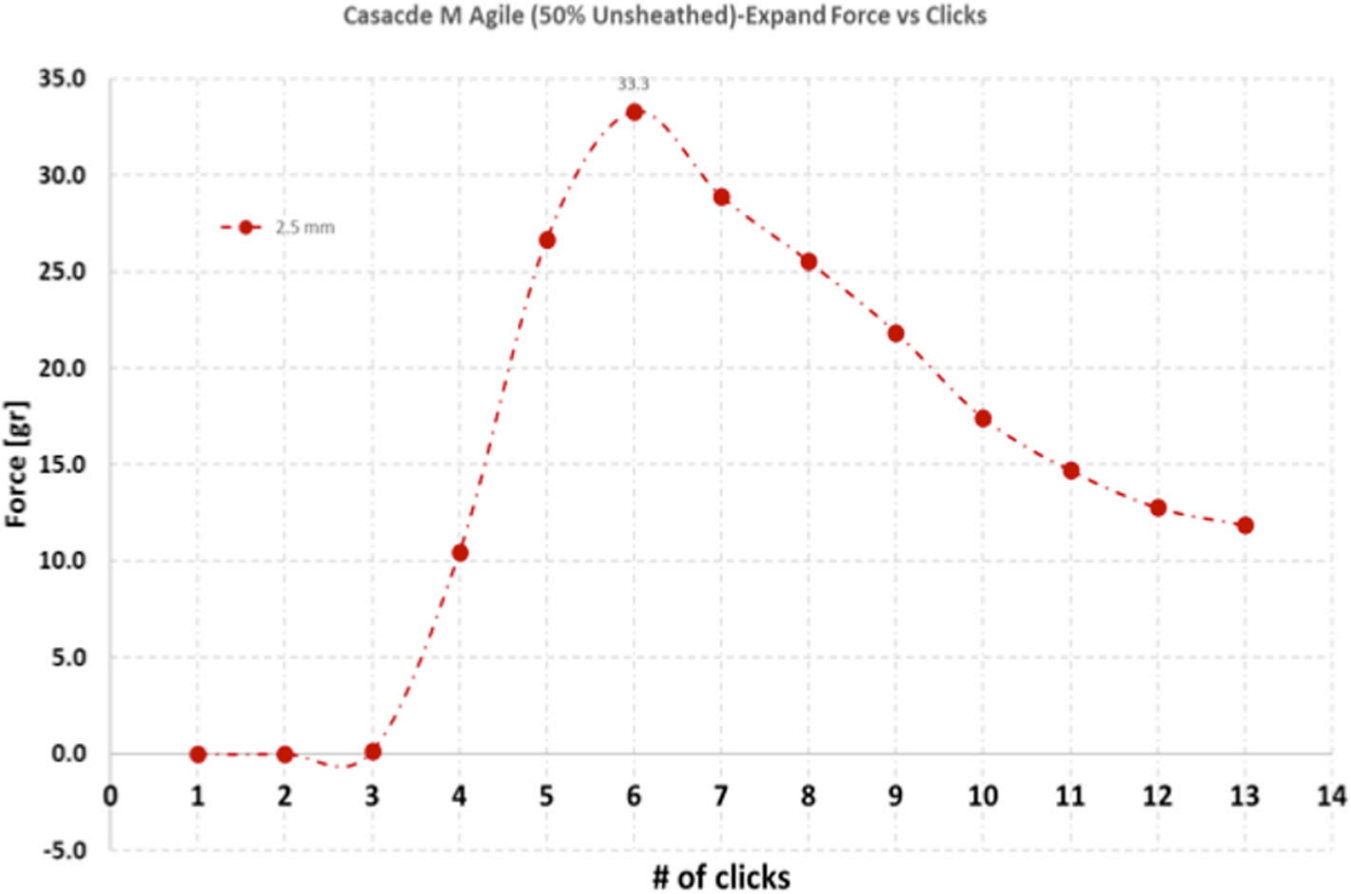
When the Cascade devices were placed in tubes of fixed diameters the forces exerted by the Cascade varied. The expansile force generated when placed into the 2.5 mm tube and partially unsheathed (50% unsheathed) demonstrated a rapid increase the force generated up to a maximum (33.3 g) followed by a rapid tapering in the force applied

In addition to testing the forces generated by the Cascade devices in the fully unsheathed state the forces generated by the devices when partially unsheathed was also tested. Figure 9 represents the forces generated when the Cascade M Agile was placed in the 2.5 mm diameter tube. This demonstrates that there is an increase in the force generated up to a maximum followed by a rapid decline in the overall force generated.

**Fig. 10:**
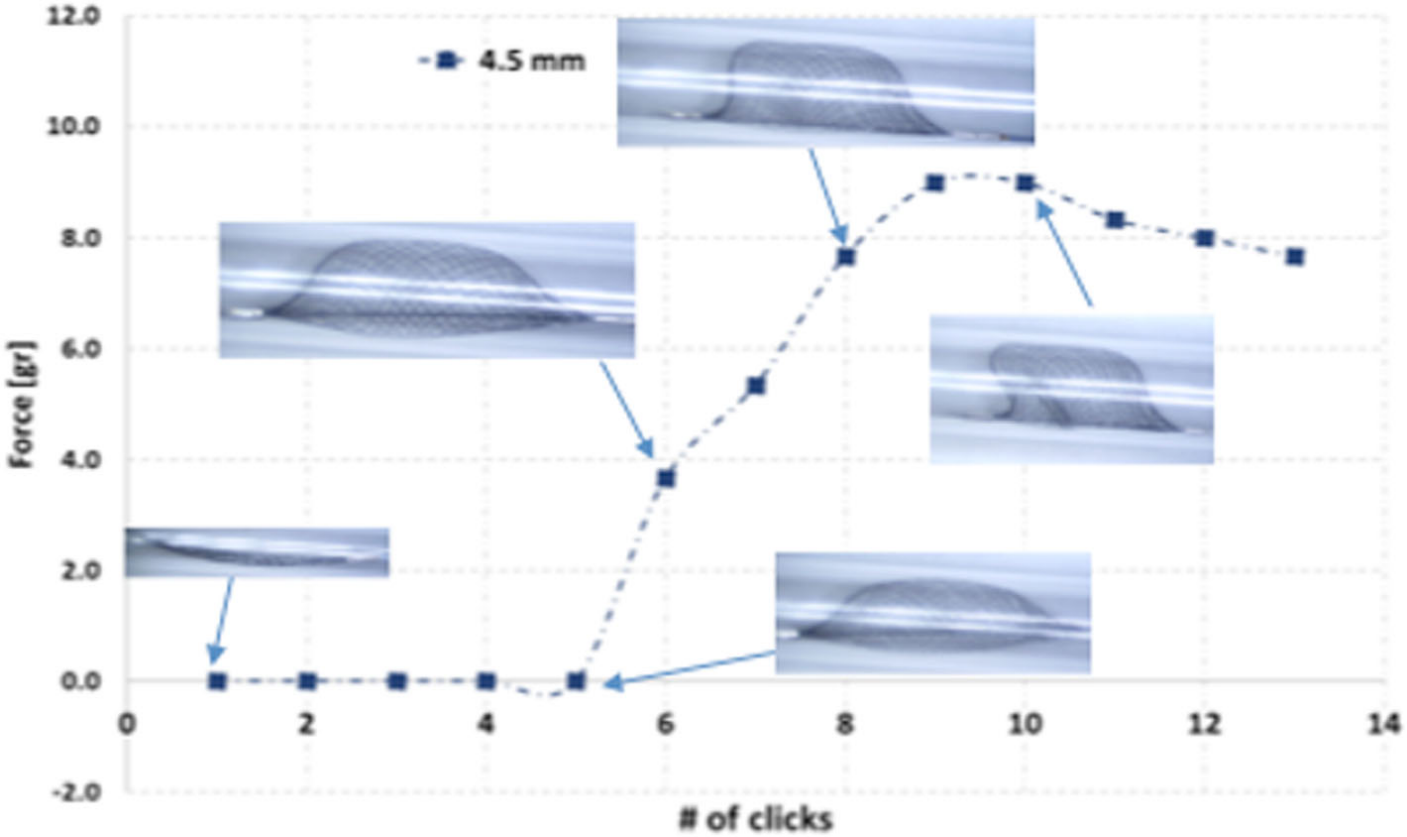
When placed into tubes below the maximal diameter of the Cascade device the force generated by the device gradually increases to a maximum. Further continued expansion of the device results in deformity of the braided structure and a decrease in the force applied to the wall of the tube

This finding was further explored by assessing the fully unsheathed Cascade L Agile in a clear plastic tube of 4.5 mm diameter (Fig. 10). Device expansion causes a gradual increase in the force exerted up to a peak, followed by a decline in the force generated. Continued expansion of the device beyond the diameter of the constraining tube results in a deformation of the braid and decline in the force generated. This was true, although to a lesser extent, when the device was also fully unsheathed. Although the maximum force generated by the fully and partially unsheathed devices were often similar, the local pressure at an individual point was likely to be higher when the device is partially unsheathed since the area of contact between the wall and the braid is reduced.

## Discussion

This is the first series to demonstrate the efficacy of the Cascade device when used to treat delayed cerebral vasospasm secondary to aSAH. The Cascade device can significantly increase the diameter of a variety of vasospastic segments including the more difficult to treat large vessels such as the ICA. The results are long-lasting with delayed angiography confirming persistent dilatation at 24 h. The bench-side analysis also demonstrates that a significantly greater force can be generated using the Cascade M than either the 4 or 6 mm Solitaire devices which would support our clinical findings that the device can be successfully used to treat cerebral vasospasm.

Bhogal et al. published the initial report on the use of stents retrievers to treat vasospasm several years ago and in this publication a variety of different devices were used all of which appeared to cause vasodilation to varying degrees. Follow-up studies performed in the patients showed a persistent dilatation. This demonstrated that the forces required to treat cerebral vasospasm were within reach of existing devices. It was noted that the success of stentplasty appeared to be higher for smaller vessels. Subsequent mathematical modelling studies corroborated this observation with the concept of a ‘dilatation threshold’ that needs to be reached in order for a sustained treatment effect (Bhogal et al., 2019). Upon reaching this threshold there would be relaxation of the vessel, due to changes in the contractile properties of the smooth muscle cells, but no disruption of the extracellular matrix. Numerous in vivo studies had demonstrated that damage to the extracellular matrix was not required for successful treatment of cerebral angioplasty (Yamamoto et al., 1992; Macdonald et al., 1995; Kobayashi et al., 1993). The concept of a dilatation threshold built upon the earlier work of Fischell who documented ‘arterial paralysis’ occurring post balloon angioplasty in both relaxed and contracted arteries, that these vessels failed to respond to the topical application of vasoconstricting agents, and that the same degree of balloon dilatation in relaxed vessels resulted in significantly less, if any, arterial paralysis compared to contracted vessels. Together these results suggested that contraction predisposed a vessel to mechanical dilatation and that the forces required to achieve this were much lower than previously thought since the extracellular matrix did not need to be disrupted to effect these changes. This idea that pre-contraction predisposes to the successful vasodilatation of vessels suggests that use of chemical vasodilators prior to mechanical vasodilatation may hinder the success of the latter. This was subsequently shown by Kwon et al. (Kwon et al., 2019) who reported higher recurrent vasospasm rates following stentplasty if vasodilators were given prior to stentplasty compared to afterwards (60% vs. 0%).

Su et al. (Su et al., 2020) recently published their results on the use of the Solitaire 6x40mm to treat vasospasm following unsuccessful IA infusion of verapamil/nicardipine. All stentplastied vessels showed calibre improvement with the average increase in vessel diameter being 106.5 ± 64.2% (range 41.8–213.6%). A greater calibre change of vessels in the posterior circulation than in the anterior circulation was noted. In our series the average increase in the diameter was approximately 300%. Our in vitro have shown that the Cascade devices are able to generate markedly greater radial forces than both the 4 and 6 mm Solitaire devices. This is important because previous studies using a variety of different stent-retrievers to treat cerebral vasospasm have shown a varied ability to treat cerebral vasospasm, which has been attributed to the radial force generated by the commercially available stent-retrievers when used in an off-label manner to treat cerebral vasospasm. The Cascade allows the operator to alter the force applied to the vessel by varying both the degree to which the device is unsheathed as well as how much the device is expanded. In larger proximal vessels full expansion of the device may be required however, in small more distal vessels the force required to cause arterial relaxation is likely to be lower and hence maximal expansion is likely not required. This was noted in several of our cases with repeated expansion and partial unsheathing was required to treat focal areas of persistent stenosis.

The most recent study, and to date the only prospective study to assess stentplasty in DCV, was published by Gupta et al. (Gupta et al., 2021). The VITAL study was a prospective, open-label, single-arm study to assess the safety and efficacy of the NeVa VS device – another stent designed specifically to target vasospasm. The primary endpoint was the ability of the NeVa VS to treat vasospasm with less than 50% spasm of the vessel, compared to baseline, following use of the NeVa VS and assessed by an independent core lab. A total of 70 patients were consented for recruitment in to the study but only 30 were treated. The mean age was 52 ± 11 years and the majority were female (86.7%). Prior to treatment the mean NIHSS score was 12.7 ± 12.8 and ASPECT score was 8.5. The MCA was the most commonly treated vessel (43/74, 58.1%). In total 34 procedures were performed with a total of 95 deployments in 74 vessel with a mean number of 1.3 ± 0.6 device deployments per vessel and mean deployment of 5.5 ± 2.2mins. Pre-treatment the degree of stenosis was 65.6 ± 14.7% and post-treatment it was 29.4 ± 19.3% with 86.5% of vessels successfully meeting the primary end-point. Subject-based procedural success was 92.2%. Retreatment of a vessel after the previous treatment with NeVa VS was low and required in in only 5/74 vessel segments (6.8%). In three of the 95 deployments (3.2%) a thrombotic event occurred that and related to the device. There were no vessels ruptures related to the NeVa VS however, in one patient where two deployments of the NeVa VS did not result in significant vasodilation a balloon angioplasty was performed with a consequent rupture of the MCA.

Non-occlusive devices offer a number of advantages relative to the use of balloons when considering cerebral vasospasm. Unlike balloons, they do not impede the anterograde cerebral blood flow which has been shown to rapidly decrease the tissue perfusion distally (Rasmussen et al., 2015). The risk of vessel rupture is likely to be lower compared to balloon angioplasty as the forces generated are significantly lower with stents. The increased familiarity with stents amongst interventional neuroradiologists is also likely to make stentplasty an inherently safer procedure compared to balloon angioplasty. More recently the growing interest and development of techniques to target distal vessel thrombectomy will also likely benefit the treatment of cerebral vasospasm in smaller distal vessels as familiarity of navigation and the use of devices in these vessels will no doubt improve. The treatment of vasospasm should aim to treat as many of the spastic segments as possible. A failure of this could inadvertently result in an arterial steal phenomenon being created such that there is preferential flow in a treated vessel that will now have lower resistance, with a precipitous drop in the flow in any vessels that remain vasospastic. This scenario was shown by Levitt et al. (Levitt et al., 2014) and in one of the cases presented by this group balloon angioplasty of the M1 segment resulted in improved flow within the MCA territory but reduced flow within the ACA territory. The easier navigability and lower profile of stents and stent-like devices compared to balloons may represent another potential advantage. In our own series, navigation of the Cascade into the ACA has proven straight-forward even in cases of severe vasospasm. Furthermore, the Cascade device may have several inherent advantages when used for cerebral vasospasm. In our experience fully unsheathing the device to treat the vasospasm initially is our preference. If after expanding the fully unsheathed device focal areas of vasospasm persist, a partially unsheathed device can be used to target the focal areas of spasm since this generates similar levels of force but over a smaller area. As was shown in our in vitro tests, the force generated by the Cascade starts to decrease if the device is expanded beyond the diameter of the vessel or constraining tube. Put another way once the vessel is dilated to its normal diameter further expansion of the device is unlikely to result in rupture due to the decrease in force applied due to braid deformation. This is the opposite of balloons whose force can be essentially continually increased until the balloon, the vessel, or both ruptures. This suggests that it would be very difficult to rupture a vessel using the Cascade device as long as one uses the device in a reasonable manner.

Given the potential advantages of stents to treat cerebral vasospasm there has been growing interest in this area. The pRELAX (phenox, Bochum, Germany) and Neva VS are the first of a new breed of retrievable stents designed with the specific intention of treating vasospasm. Early results of the pRELAX devices have recently been presented with promising results and publications are awaited. It is worthwhile noting that only the pRELAX device has been given a CE mark for treatment of cerebral vasospasm and use of other devices is currently off-label.

Our study is limited by its retrospective design and low number of cases included in the study.

## Conclusion

The Cascade is a safe and highly effective device when used to treat cerebral vasospasm. Larger studies are required to validate our initial results.

## Data Availability

There is no additional data or material available.

## Abbreviations

aSAH: Aneurysmal Subarachnoid Haemorrhage
ACA: Anterior Cerebral Artery
COF: Chronic Outward Force
CTA: Computer Tomography Angiography
CT: Computer Tomography
DCV: Delayed Cerebral Vasospasm
ICA: Internal Carotid Artery
IU: International Units
MCA: Middle Cerebral Artery
RCT: Randomised Controlled Trial
WFNS: World Federation of Neurosurgical Societies
